# Off-label use of muscular VSD device for closure of a rare congenital portosystemic shunt

**DOI:** 10.1186/s43044-024-00467-5

**Published:** 2024-03-23

**Authors:** Hala Mounir Agha, Shady Mashoor, Mohamed Ghobashy, Antoine AbdelMassih, Hanya Gaber, Hanaa El-Karaksy

**Affiliations:** 1 Pediatric Cardiology Unit, Pediatrics’ Department, Faculty of Medicine, Cairo University, P.O BOX: 12411, Cairo, Egypt; 2 Interventional Radiology Unit, Radiology Department, Faculty of Medicine, Cairo University, Cairo, Egypt; 3 Faculty of Medicine, Cairo University, Cairo, Egypt; 4 Pediatric Hepatology Unit, Faculty of Medicine, Cairo University, Cairo, Egypt

**Keywords:** CPSS, Unexplained pulmonary hypertension, mVSD device

## Abstract

**Background:**

Congenital portosystemic shunt (CPSS) is a vascular malformation in which portal blood drains toward the systemic circulation, leading to pulmonary hypertension.

**Case presentation:**

A 10-year-old patient was brought for evaluation because of dyspnea on exertion. Echocardiography revealed a pulmonary hypertension of 75 mmHg, and multi-slice CT angiography revealed the presence of a CPSS. Closure was finally implemented using a muscular ventricular septal defect device. Follow-up of the patient revealed a gradual decline in pulmonary hypertension.

**Conclusions:**

CPSS is an overlooked cause of reversible pulmonary hypertension (PH). Closure of such lesions and reversal pulmonary hypertension are possible via catheterization. The preferred device type depends largely on the intervening team. Plugs are the first choice for interventional radiologists, while ventricular and atrial septal occluder devices and duct occluders are preferred by pediatric cardiologists.

## Background

Congenital portosystemic shunt (CPSS), also known as Abernethy malformation, is a rare vascular malformation in which portal blood drains into the systemic circulation, eluding the liver [1, 2].

According to the physiopathological theory, when vasoactive substances present in the intestinal circulation (e.g., serotonin, histamine, estrogen, glucagon) bypass the liver without being metabolized and pass through a CPSS, this results in pulmonary arterial hypertension (PAH) caused by the induction of long-lasting pulmonary vasoconstriction [3, 4].

CPSS has a wide spectrum of manifestations that can occur at any point in an individual's life, although asymptomatic cases that are incidentally detected on imaging are also common. In children, long-term portosystemic shunting leads to the most prominent manifestations such as hepatopulmonary syndrome, pulmonary hypertension, and hepatic encephalopathy. Even before birth, a disruption in fetal venous circulation caused by shunting may result in reduced liver perfusion and signs of intrauterine growth restriction, without the presence of hypoxia, maternal infections, and/or chromosomal abnormalities. Neonatal cholestasis and galactosemia are among the complications that may arise and should be distinguished from other congenital defects such as biliary atresia and metabolic disorders that may coexist [5, 6].

## Case presentation

### Case description

**Fig. 1:**
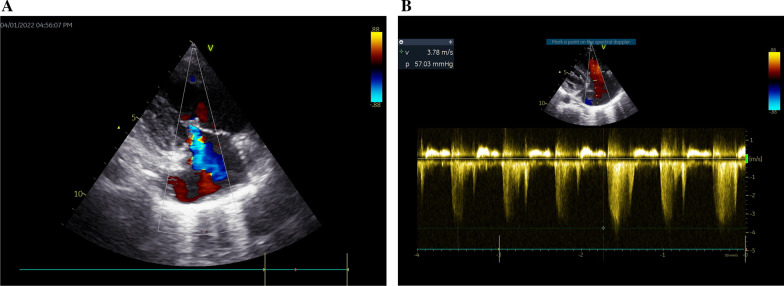
**A** Tricuspid regurgitant jet. **B** Continuous wave Doppler gradient across the across the tricuspid regurgitant jet

A 10-year-old patient was brought for evaluation because of easy fatigability and dyspnea on exertion, with no clinically appreciable cyanosis. Patient anthropometric measurements showed mainly failure to thrive with a preserved stature centile; weight was 19 kg (< 5th percentile for age and sex), while stature was 125 cm (25th percentile for age and sex). Pulmonary hypertension was evident in the accentuated pulmonary component of the 2nd heart sound. Significant right atrial and ventricular dilatation were elicited by echocardiography, along with an estimated systolic pulmonary arterial pressure of 62–67 mmHg, obtained via tricuspid regurgitant jet (Fig. 1A–B). Left-to-right shunts and underlying cardiac abnormalities were not observed.

Before concluding the diagnosis of primary pulmonary hypertension, multi-slice CT angiography was performed, which revealed the presence of CPSS.

**Fig. 2:**
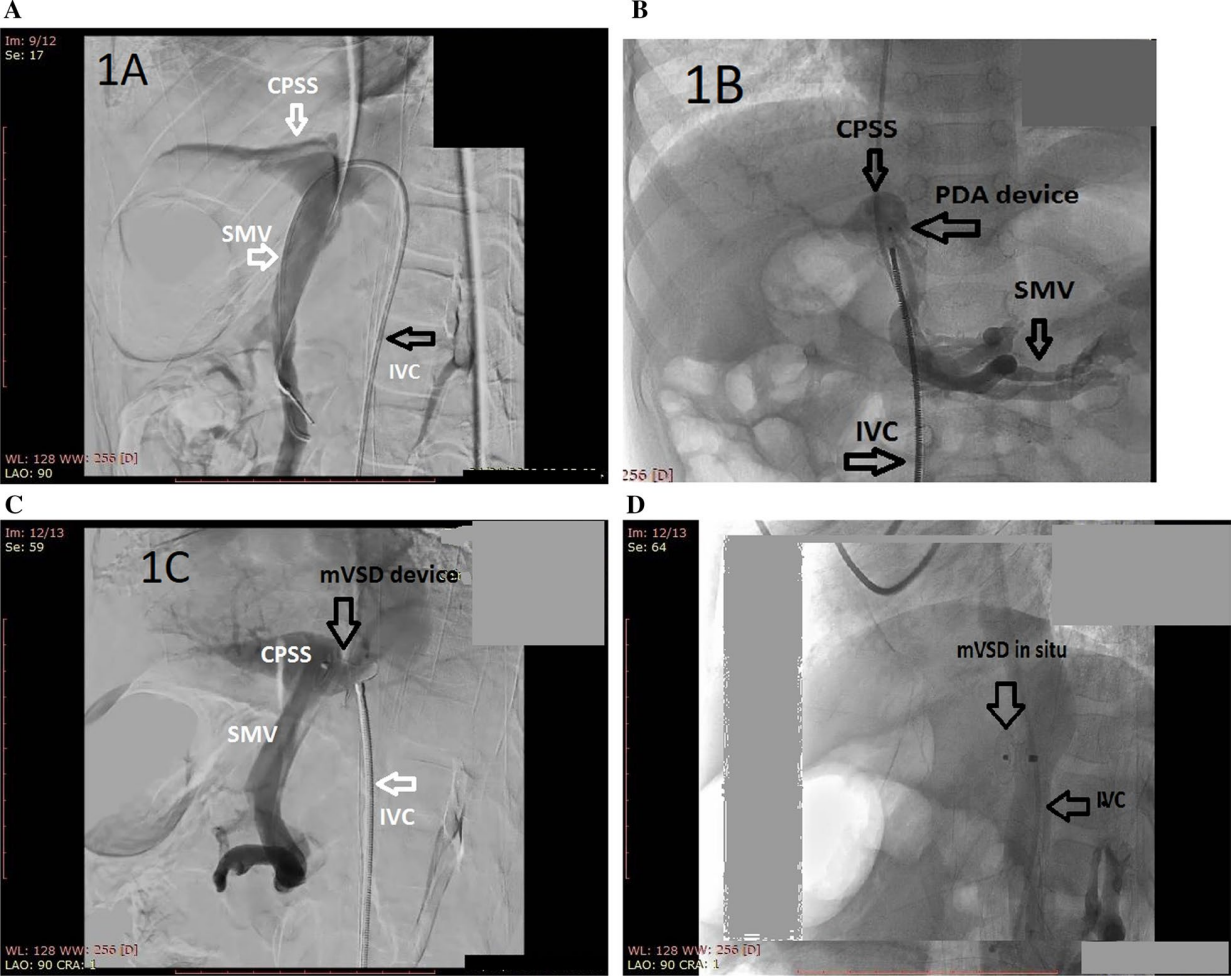
**A** IVC angiogram showing CPSS. *CPSS* Congenital portosystemic shunt, *IVC* Inferior vena Cava, *SMV* Superior mesenteric vein. **B** Prolapsed ADO device. *ADO* Amplatzer duct occluder, *CPSS* Congenital portosystemic shunt, *IVC* Inferior vena Cava, *PDA* Patent ductus arteriosus, *SMV* Superior mesenteric vein. **C** Muscular Device placed across the fistula before deployment *CPSS* Congenital portosystemic shunt, *IVC* Inferior vena Cava, *SMV* Superior mesenteric vein. **D** Muscular Device placed across the fistula after deployment. *IVC* Inferior Vena Cava, *mVSD* Muscular ventricular septal defect

**Fig. 3:**
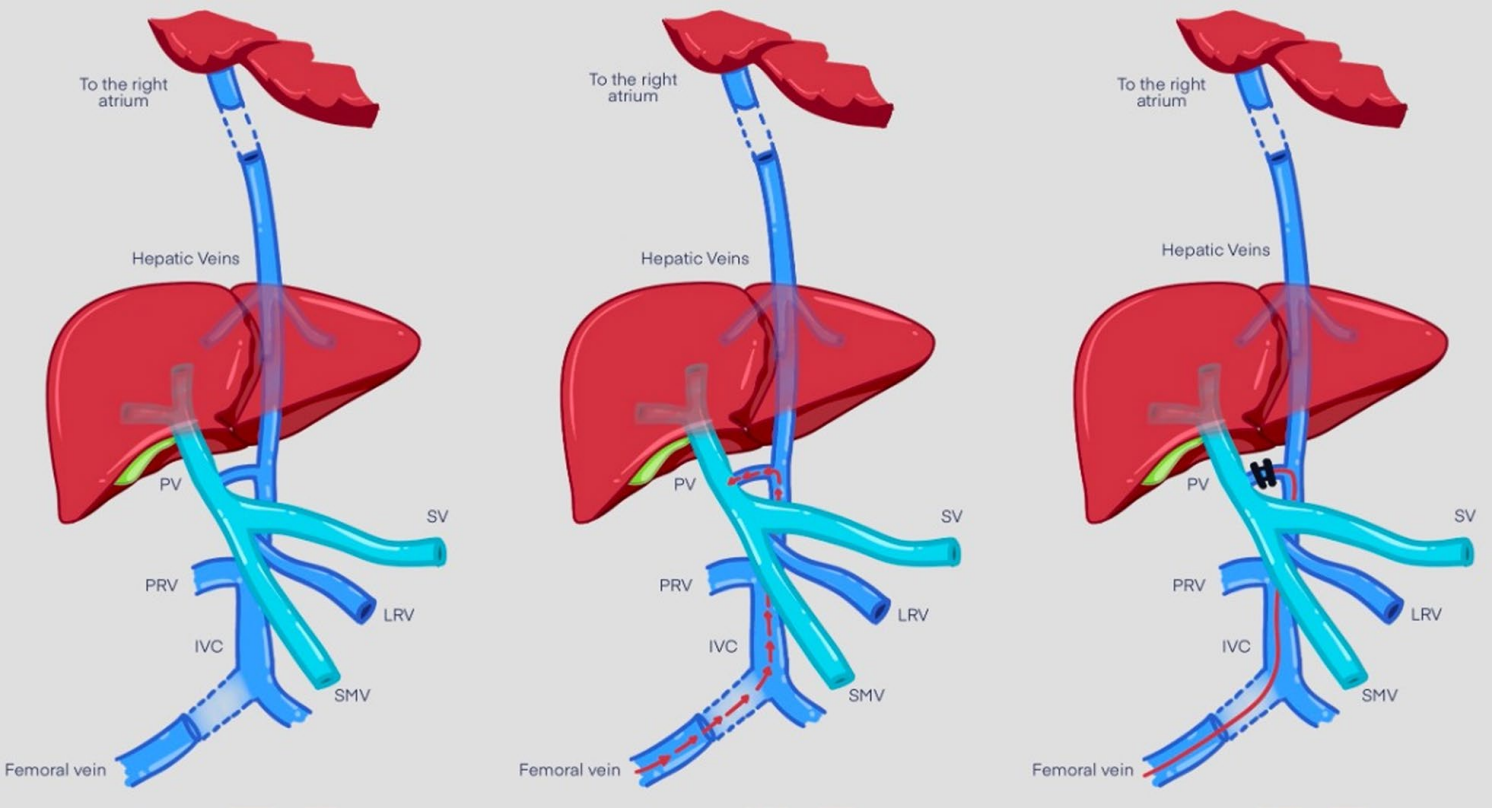
Diagram of the approach used for closure of the CPSS. Parts: Part 1 showing the fistula/Part 2: Shows the pathway used for closure, Part 3 shows the device across the fistula. *IVC* Inferior vena cava, *LRV* Left renal vein, *PV* Portal vein, *SV* Splenic vein, *SMV* Superior mesenteric vein.

Transcatheter closure of the portosystemic shunt was then attempted. For this purpose, a 6 Fr femoral vein, a 5 Fr Internal jugular vein access, and a 5 Fr femoral arterial access were prepared. Invasive hemodynamics were observed, which revealed elevated pulmonary vascular resistance. Inferior vena cava (IVC) injection revealed a previously identified fistulous malformation between it and the portal vein. It is conical in shape, with the largest diameter at the vena cava side (approximately 8 mm). The first attempt was performed using a Lifetech ductal occluder I DOI (8/6) device (Lifetech Scientific, Shenzhen, China) for shunt closure, but unfortunately, it was unstable in position and slipped into the IVC. A decision was made to close it using a Lifetech muscular mVSD device with a size of 8 mm (Lifetech Scientific, Shenzhen, China). A 4F Judkins Rt catheter under Terumo wire guidance in the IVC was used to cross the fistula to the portal vein. The Terumo wire was withdrawn and replaced by a stiff wire 0.035 × 260 cm to secure the position in the portal vein, which was then withdrawn and replaced by a multipurpose 5 Fr catheter to support the delivery system of the muscular device. The device was loaded onto a 7 Fr Lifetech delivery system, and the entire system was advanced along the guidewire and multipurpose catheter. Once part of the portal vein adjacent to the fistula was reached, the device was released progressively to seal the CPSS. Repeated injections revealed adequate closure of the fistula. (Figs. 2A–D and 3).

## Discussion of the case

Due to the increased levels of humoral substances in the lung tissue, chronic pulmonary vasoconstriction can occur in patients with CPSS. Our patient presented with difficulty breathing due to severe pulmonary hypertension, in addition to hyperammonemia due to concomitant liver dysfunction [4].

Kuo and colleagues performed the first endovascular closure of a portosystemic shunt in 2010. They used a three-stage approach due to associated portal vein hypoplasia. Subsequently, several studies have been published [7].

It is noticeable from the cases summarized in our table that interventional radiologists rely on vascular plugs in the closure of this malformation, whereas pediatric cardiologists perform most of their procedures using duct, atrial septal, and ventricular septal occluders. Vascular plugs are primarily used to treat extracardiac defects. Perhaps this is the reason why they are rarely used by interventional cardiologists and thus rarely available in pediatric cardiac catheter laboratories. This probably explains the bias in the devices used by different teams to close the same defect.

Table 1 shows reports where the endovascular approach was used for CPSS closure [7–13].

**Table 1:** Examples of endovascular approach to congenital portosystemic shunts

Year	Type of publication/number of patients	Author	Service provided by	Presenting manifestation	Age	Size of the shunt	Type of occluding device
2010	Case report/1	Kuo et al	Interventional radiology	Progressive cyanosis	11 years	14 mm	Graded closure using covered stents to allow growth of portal veins followed by complete closure by vascular plug
2012	Case report/1	Passalacqua et al	Interventional Radiology	Progressive cyanosis	3 years	10 mm	Vascular plug 10 mm
2013	Case Series/4	Bruckheimer et al	Pediatric cardiology	Four patients all presenting with hepatic encephalopathy	2.5 years, 4 years, 8 and 10 years	Not reported	Graded closure using covered stents followed by duct occluder
2017	Case report/1	AlHarbi et al	Pediatric Cardiology and Interventional radiology	Hepatic encephalopathy	1 month-old	4 mm	Duct occluder 12 × 6 mm
2017	Case series/2	Tomiyama et al	Pediatric Radiology	Two patients presenting with Hepatic encephalopathy	75 and 83 years old	Not reported	22- and 14-mm vascular plugs, respectively
2021	Case report/1	Facas et al	Interventional Radiology	Asymptomatic: during routine screening of liver functions before isotretinoin therapy for acne	15 years	16 mm	16 mm atrial septal occluder followed by 14 mm vascular plug
2021	Case report/1	Shnayder et al	Interventional Radiology	Hepatic encephalopathy	2 years	14 mm	18 mm patent foramen ovale device
2022	Case series/21	Koneti et al	Pediatric cardiology	11 with progressive cyanosis 6 with pulmonary hypertension 2 with hepatic encephalopathy 2 with pulmonary hypertension and progressive cyanosis	0.45 to 19 years	7–16 mm	Ten with vascular plugs Six with muscular ventricular septal defect (VSD)device Four by septal occluder Graded closure using covered stents followed by Muscular VSD One by duct occluder

A very important consideration to be taken into consideration, during and after closure, is the possible hypoplasia of intrahepatic pulmonary veins. This hypoplasia is either managed with staged repair or with close follow-up every 3–6 months for any signs of portal hypertension and serial ammonia measurements after shunt closure. Nevertheless, prolonged intrahepatic hypoxia can stimulate neoplastic activity and nodule formation; therefore, experts recommend annual abdominal ultrasound to detect any liver neoplasm [6].

## Conclusions

When dealing with PAH patients, CPSS should be ruled out, despite its rarity, especially in the pediatric field. The damage caused by PAH can be reversed by transcatheter shunt closure. The endovascular approach has supervened classic surgical ligation, and off-label use of intracardiac devices is increasingly being implemented.

## Data Availability

Catheter images have been submitted with manuscript.

## Abbreviations

ADO: Amplatzer duct occluder
CPSS: Congenital portosystemic shunt
EPSS: Congenital extrahepatic portosystemic shunt
IVC: Inferior vena cava
PAH: Pulmonary arterial hypertension
PFO: Patent foramen ovale
VSD: Ventricular septal defect
